# Manual of Human Anatomy, &c.

**Published:** 1838-07

**Authors:** 


Art. XIV.—*Handbuch der Menschlichen Anatomie.* Von C. F. T. Krause, m.d., Professor der Anatomie; an Ersten Bandes, dritte Abtheilung.—*Hannover*, 1838. 8vo.
*Manual of Human Anatomy, &c.* By Dr. Krause.

We noticed with approbation the preceding parts of this work in our Fifth number. The present Part completes the first volume, which comprehends the whole of the general and special anatomy of the adult. The two books now before us, containing the anatomy of the vascular and nervous systems, are composed in the same minute and careful manner as the preceding; and we repeat our former statement as to the very great merits of the whole work. We are happy to find that Dr. Krause's manual will shortly be presented to the English reader translated by Mr. Morley.

